# Severe Uncontrolled Asthma: A Longitudinal Retrospective Study Illustrating the Experience of the Pulmonology Clinic of Târgu-Mureș, Romania

**DOI:** 10.3390/jcm13216582

**Published:** 2024-11-01

**Authors:** Edith-Simona Ianoși, Dragoș Huțanu, Mara Andreea Vultur, Hédi-Katalin Sárközi, Delia-Liana Rachiș, Gabriela Jimborean

**Affiliations:** 1 Department of Pulmonology, University of Medicine and Pharmacy, Science and Technology “George Emil Palade” of Târgu Mureș, 540139 Târgu Mureș, Romania; edith.ianosi@umfst.ro (E.-S.I.); mara.vultur@umfst.ro (M.A.V.); hedi.balog@umfst.ro (H.-K.S.); gabriela.jimborean@umfst.ro (G.J.); 2 Pulmonology Clinic, Mureș County Clinical Hospital, 540011 Târgu Mureș, Romania; deliarachis@yahoo.ro

**Keywords:** asthma, biologic therapy, severe uncontrolled asthma, allergic diseases

## Abstract

**Introduction:** Severe uncontrolled asthma (SUA) affects approximately 5% of asthma patients, leading to frequent exacerbations, reduced lung function, and lower quality of life. Recent biologic therapies target specific inflammatory pathways, offering new options for SUA. **Objective**: This study aimed to evaluate clinical characteristics, treatment outcomes, and biomarkers in patients with SUA treated with biologics (Omalizumab, Benralizumab, and Dupilumab) at our clinic. **Material and Methods:** A six-month retrospective longitudinal study was conducted on 28 patients aged 36–83 years with SUA. Patients were divided into three groups: Omalizumab (*n* = 4), Benralizumab (*n* = 18), and Dupilumab (*n* = 6). Lung function tests and biomarkers such as eosinophil and IgE levels were measured over 3-month periods (T0, T1, and T2). Asthma control was assessed using asthma control tests (ACT), and non-parametric statistical methods were applied. **Results:** The median patient age was 64 years, with 75% showing elevated eosinophil counts (>300 cells/µL). Benralizumab significantly improved lung function (*p* < 0.05) and ACT scores (*p* < 0.001), reducing eosinophil counts to zero (*p* < 0.001). Patients on Dupilumab and Omalizumab showed improved asthma control (*p* < 0.05) and reduced exacerbations, albeit to a lesser extent (*p* > 0.05). **Conclusions:** Biologics, particularly Benralizumab and Dupilumab, improved asthma control, lung function, and quality of life in SUA patients, with improved ACT scores and spirometry values. Some patients remained poorly controlled, emphasizing the need for personalized treatment and regular biomarker monitoring. Multidisciplinary management and lifestyle changes are critical for better outcomes in SUA.

## 1. Introduction

Asthma is a chronic respiratory disease marked by inflammation and hyperreactivity of the airways, leading to symptoms like wheezing, shortness of breath, chest tightness, and coughing [1]. While most asthma patients achieve symptom control with standard therapies, approximately 5% of individuals suffer from severe asthma, a condition that remains difficult to manage despite high-dose inhaled corticosteroids (ICS) and additional controller therapies [2]. Severe asthma is associated with frequent exacerbations, a higher risk of hospitalization, reduced lung function, and diminished quality of life [1,2]. Moreover, it accounts for a disproportionate share of asthma-related healthcare costs and mortality [1]. This article provides an overview of patients with severe uncontrolled asthma (SUA) admitted in the Clinic of Pneumology from Târgu-Mureș, Romania, for 6 months, emphasizing the latest treatment approaches, clinical outcomes, and emerging therapies, considering the slow introduction of biologic therapy for SUA in Romania and the highly maintained reticence of prescription in our country.

### 1.1. Immunobiological Treatments: Mechanisms and Targets

Immunobiologicals, or biologics, are a class of drugs that specifically target molecules involved in the immune response [2]. In the context of asthma, these treatments are designed to interrupt the pathways that lead to chronic airway inflammation.

### 1.2. Anti-IgE Therapy

Omalizumab, a monoclonal antibody targeting immunoglobulin E (IgE), was the first biologic approved for asthma treatment. IgE plays a critical role in the allergic response and, by inhibiting its activity; Omalizumab reduces the frequency of exacerbations and improves asthma control in patients with allergic asthma [3].

### 1.3. Anti-IL-5 Therapy

Interleukin-5 (IL-5) is a cytokine essential to the survival and activation of eosinophils, which are key players in the inflammatory process of asthma. Mepolizumab, Reslizumab, and Benralizumab are monoclonal antibodies that target IL-5 or its receptor. These biologics have been shown to significantly reduce exacerbation rates and the need for oral corticosteroids in patients with eosinophilic asthma [4,5,6].

### 1.4. Anti-IL-4/IL-13 Therapy

IL-4 and IL-13 are cytokines involved in the production of IgE and the promotion of airway hyperresponsiveness and mucus production. Dupilumab, a monoclonal antibody that blocks the IL-4 receptor alpha, inhibits both IL-4 and IL-13 signaling. Clinical trials have demonstrated that Dupilumab reduces exacerbations and improves lung function in patients with moderate-to-severe asthma, particularly those with elevated eosinophil levels or high fractional exhaled nitric oxide (FeNO) [7,8].

### 1.5. Anti-TSLP Therapy

Tezepelumab is a monoclonal antibody that targets thymic stromal lymphopoietin (TSLP), an epithelial cytokine that plays a key role in the initiation of allergic inflammation. By blocking TSLP, Tezepelumab can reduce exacerbations in a broad population of patients with severe asthma, including those who do not have elevated eosinophil counts [9].

### 1.6. Clinical Benefits and Impact on Quality of Life

Immunobiological treatments have revolutionized the management of severe asthma. These therapies are particularly beneficial for patients who have specific biomarkers, such as elevated blood eosinophils or high IgE levels, which indicate a particular type of inflammation driving their disease. By targeting the underlying mechanisms of inflammation, biologics have been shown to reduce the frequency of asthma exacerbations, improve lung function, and decrease the need for oral corticosteroids, which are associated with significant side effects when used long-term [10].

Furthermore, patients receiving biologics report improvements in quality of life as they experience fewer asthma symptoms and are able to engage in daily activities with greater ease. The ability to reduce exacerbations also translates to fewer emergency room visits and hospitalizations, alleviating the burden on healthcare systems.

## 2. Material and Methods

### 2.1. Study Design

A longitudinal retrospective observational study was conducted over 6 months in the Clinic of Pneumology from Târgu-Mureș, Romania, to evaluate the clinical characteristics, treatment outcomes, and biomarkers in patients with severe asthma. This study was approved by the Institutional Review Board (no.13871/09.09.2024), and informed consent was obtained from all participants.

This study included 28 patients with ages ranging from 36 to 83 years old, diagnosed with severe uncontrolled asthma (SUA) according to the European Respiratory Society/American Thoracic Society (ERS/ATS) guidelines [11]. Patients with other chronic respiratory conditions (tuberculosis, lung cancer, idiopathic fibrosis, COPD) or those noncompliant with the treatment regimen were excluded.

Criteria for biologic treatment introduction were respected as follows: for Benralizumab, age over 18 years-old, peripheral blood eosinophils ≥300 cells/μL at the initiation of treatment or ≥150 cells/μL in patients treated intermittently or continuously with oral corticosteroids (OCS) at ≥8 mg/day (8 mg prednisone or equivalent to 6 mg methylprednisolone), and asthma management prescribed by a specialist physician with a minimum follow-up period of 6 months, including treatment with high-dose inhaled corticosteroids, as recommended by GINA, combined with a long-acting beta-2 agonist for a minimum of 6 months (with correct inhaler technique and adherence to treatment confirmed by the attending physician) and proper management of comorbidities (e.g., chronic rhinosinusitis, gastroesophageal reflux, psychological disorders) or other conditions (e.g., cigarette smoking); for Omalizumab, adults, adolescents, and children over the age of 6, diagnosis of severe asthma, according to the recommendations of GINA, confirmed IgE-mediated allergy demonstrated by at least one of the following (including history): positive skin prick test for at least one perennial aeroallergen or presence of specific IgE antibodies for at least one perennial aeroallergen (above the laboratory threshold level), optimized asthma management by a specialist physician with a minimum follow-up period of 6 months, which includes treatment with high-dose inhaled corticosteroids, according to GINA recommendations, in combination with a long-acting beta-2 agonist for at least 6 months (with correct inhaler technique and adherence confirmed by the attending physician) and proper management of comorbidities (e.g., chronic rhinosinusitis, gastroesophageal reflux, psychological disorders) or other conditions (e.g., cigarette smoking), together with lack of asthma control as defined by GINA guidelines, indicated by one of the following: poor symptom control (ACT score < 20 or ACQ score > 1.5) or frequent exacerbations (≥2/year) requiring oral corticosteroids or severe exacerbations (≥1/year) requiring hospitalization; for Dupilumab, adults and adolescents with severe asthma aged 12 years and over and children with severe asthma aged 6 to 11 years, patients with inadequately controlled severe asthma (with exacerbations in the past year) on high-dose inhaled corticosteroids (ICS) or who are controlled only with oral corticosteroids (OCS) (either the lowest possible intermittent dose of OCS or corticosteroid-dependent patients) who, upon assessment of their severe asthma phenotype, have a Th2 type asthma and, according to the GINA recommendations, still present with blood eosinophils ≥ 150 cells/μL and less than 1500 cells/μL or FeNO ≥ 20 ppb or eosinophils in sputum ≥ 2% or allergic asthma indicators (patients sensitized to an aeroallergen with IgE > 30 IU/mL–1300 IU/mL) or requirement for maintaining OCS to ensure control and prevent frequent exacerbations (≥2/year) or requiring oral corticosteroids or having severe exacerbations (≥1/year) requiring hospitalization, asthma management prescribed by a specialist physician, with a minimum follow-up period of 6 months, which includes treatment with high-dose inhaled corticosteroids, according to GINA recommendations, in combination with a long-acting beta-2 agonist (correct inhaler technique and adherence to treatment confirmed by the attending physician), together with proper management of comorbidities (e.g., chronic rhinosinusitis, gastroesophageal reflux, psychological disorders) or other conditions (e.g., cigarette smoking or vaping) and lack of asthma control as defined by GINA guidelines, indicated by one of the following: poor symptom control (frequent symptoms or frequent use of symptom-relief therapy, asthma-limited activity, nighttime awakenings due to asthma) or frequent exacerbations (≥2/year) requiring oral corticosteroids or severe exacerbations (≥1/year) requiring hospitalization.

Patients were assigned to the corresponding treatment variant, considering their SUA phenotype, after pursuing Lung function evaluation through spirometry and blood analysis, with emphasis on eosinophilic counts and IgE values. Therefore, treatment with biologics was initiated as follows: Omalizumab therapy was initiated in 4 (14.2%) patients, Benralizumab therapy in 18 (64.2%) patients, and Dupilumab therapy in 6 (21.4%) patients, due to presence of specific SUA phenotypes.

### 2.2. Data Collection

Data were collected using patient records, including demographic details, asthma history, risk factors, comorbidities, medication usage, and symptoms and presence of exacerbations. Lung function tests (spirometry), blood eosinophil counts, and immunoglobulin E (IgE) levels were measured. Additionally, patients were assessed for asthma control using the asthma control test (ACT). Patients were evaluated over 3 time periods (T0—initial evaluation; T1—3 month evaluation; T2—6 months evaluation).

Statistical analysis was realized with IBM SPSS Statistics version 26.0.0.0, where the distribution of quantitative data was tested through histograms, Q-Q plots, and finally through the Shapiro–Wilk test for normality, confirming the presence of non-parametrical data. Therefore, all results referring to quantitative data were expressed as the median (Q25-Q75). Qualitative data were analyzed using frequencies, with results expressed in *n* (%). Differences between the study groups were analyzed through the Mann–Whitney test for independent samples, Wilcoxon test, or Friedman’s test accordingly for related samples, setting the significance limit at α = 0.05.

## 3. Results

### 3.1. Demographics and Clinical Characteristics

Of the 28 patients with severe uncontrolled asthma (SUA), the median age was 64 (54–67) years old. Nineteen (67.9%) patients came from urban environments. Sixteen (57.1%) were female, with a median age of 56 (46–65) years. Clinical history mainly consisted of dyspnea (26, 92.9%), wheezing (10, 35.7%), and productive coughing (13, 46.4%). All patients had SUA, and most of them (27, 96.4%) were staged in GINA V stage of asthma. Twenty-seven (96.4%) patients experienced two or more exacerbations requiring oral corticosteroids (OCS) in the previous year (before initiation of the biologic therapy).

Regarding comorbidities, patients mostly presented with an associated history of arterial hypertension (22, 78.6%), ischemic heart disease (11, 39.3%), pulmonary fibrosis (6, 21.4%), and bronchiectasis (7, 25%). Only 5 (17.9%) had heredocolateral antecedents of atopy, but 15 (53.6%) reported in their personal medical history signs of allergies, mostly presenting allergic rhinitis. A total of 8 (17.8%) presented with a history of repeated surgical ENT interventions with regard to rhinitis and nasal polyposis treatment. Occupational exposure was found in 14 (50%) patients, and 16 (57.1%) were former smokers.

The median duration of asthma was 17 years. Despite high-dose ICS and long-acting beta-agonists (LABAs) (double therapy) or ICS–LABA plus tiotropium (triple therapy), patients showed poor control of their asthma. Interventions like oxygen therapy or nebulization, as needed, were required in 13 (46.4%) and 6 (21.4%) patients, respectively.

### 3.2. Biomarkers and Lung Function

The median white blood cell count value at T0 was 7925 (6758–9765). Elevated blood eosinophil counts (>300 cells/µL) were observed in 21 (75%) patients, indicating eosinophilic inflammation and phenotype. The mean pre-bronchodilator FEV1 was 56.70 (46.52–65.60) of the predicted value, with an inverse correlation between FEV1 and blood eosinophil count (r = −0.309; *p* = 0.11).

### 3.3. Asthma and Quality of Life Evolution at T1 and T2

Patients’ evolution at T1 and T2, respectively, was assessed for each biological treatment in order to observe the presence of a positive effect on lung function or other biomarkers.

Over the three time periods, patients treated with Omalizumab (Table 1) presented a significant increase in their ACT scores (*p* < 0.05), proving a high increase in their quality of life and higher lung function values (FVC, FEV1, MEF50) and revealing a positive impact of this therapy, albeit remaining over the significance threshold. Furthermore, IgE values lowered significantly, proving its efficacy.

On the other hand, patients with Benralizumab therapy (Table 2), evaluated at T0, T1, and T2, presented significantly higher values of FVC (*p* = 0.04) and ACT scores (*p* < 0.001) over the three time-frames, showing its impact on both quality of life and lung function. Eosinophil counts reduced drastically to values of 0, (*p* < 0.001), emphasizing the impact of this drug on the eosinophilic component of asthmatic patients.

In the same manner, patients treated with Dupilumab therapy (Table 3) presented increased lung function values after 6 months (but *p* > 0.05) and significantly higher ACT scores (*p* < 0.01), reflecting a positive impact on the quality of life of these patients.

Only two patients from all included patients (7.14%) in the study experienced exacerbations that required hospitalization due to viral infection during the follow-up period.

## 4. Discussion

This study highlights the heterogeneity of severe asthma and underscores the importance of personalized medicine in its management. Severe asthma is a complex disease that involves multiple phenotypes and endotypes, necessitating a tailored approach to treatment [12].

Eosinophilic inflammation, indicated by elevated blood eosinophil counts, was prevalent among the study population, corroborating findings from other studies that suggest a significant role for eosinophils in severe asthma [13].

Smoking still remains an important risk factor in the evolution of the SUA in our study, with values much higher than the Romanian general population (34%) [14] and even than the European Community (28%) [15].

Biomarkers such as blood eosinophils are increasingly used to identify patients who are likely to benefit from targeted therapies. For instance, the introduction of biologic agents like Benralizumab, Dupilumab, Omalizumab has revolutionized the treatment landscape for patients with severe eosinophilic asthma, resulting in better disease control and reduced exacerbations [2,3,6].

In this study, patients who received biologic therapy demonstrated marked improvements in asthma control, as measured via ACT scores, and a significant reduction in exacerbation frequency, consistent with the findings of other clinical trials [4,6,16].

Benralizumab therapy showed the highest impact on our patients, through high reduction of peripheral eosinophil counts, high improvement of ACT scores, and lung function improvements, with the impact also being backed by the recent literature [17,18].

Dupilumab therapy brings improvement to the treatment of SUA patients, but due to the small population size in this study, significance may be affected. The literature also presents its high impact benefits in treating severe asthma, even in comparison with other biologics such as Omalizumab or Benralizumab, which also present a positive impact on SUA patients [19,20,21].

Despite these findings, a proportion of patients remain poorly controlled, even with the use of biologics, presenting exacerbations that lead to hospitalization and add-on adjuvant treatments. This matter underscores the need for ongoing research to identify additional biomarkers and novel therapeutic targets. For example, there is growing interest in the role of other inflammatory pathways, such as those involving interleukin-13 (IL-13) and thymic stromal lymphopoietin (TSLP), which may provide new avenues for treatment [13,22]. Furthermore, early implementation of biologic treatment in patients that meet the specific criteria should be considered more frequently in order to allow for prevention of disease progression and exacerbation.

The correlation between lung function and biomarkers further supports the need for the regular monitoring of patients with severe asthma. The moderate inverse correlation between FEV1 and blood eosinophil counts observed in this study suggests that persistent eosinophilic Th2 inflammation may contribute to progressive airway remodeling and decline in lung function [23]. Regular assessment and adjustment of therapy based on biomarker levels could help mitigate this risk.

This study’s findings are consistent with the current understanding of severe asthma but also highlight the challenges in achieving optimal control. The heterogeneous nature of the disease, coupled with the variability in responses to treatment and the chronic risk factors for severity, which are difficult to eliminate, suggests that a one-size-fits-all approach is inadequate. Instead, a more individualized strategy, incorporating regular biomarker monitoring and the use of targeted therapies, is essential for improving outcomes in severe asthma [3,12].

However, our study population was small; hence, there is the possibility that our results may lack of significance. Further studies in this area, carried out on higher numbers of patients, should be considered in order to confirm the relevant findings regarding the control and treatment of SUA.

## 5. Conclusions

Severe asthma remains a challenging condition even if the percentage of asthma sufferers in this category is relatively low. The number of eligible patients would have been higher if they had accepted the treatment according to the recommendations of the specific guidelines.

The identification of distinct phenotypes has significantly improved outcomes for our patients. SUA requires a multifaceted approach for management improvement.

Smoking and occupational exposure played a major role in the difficult management of SUA and in its severity; therefore, smoking cessation education and occupational hygiene have to be improved in our society. Chronic rhinitis and nasal polyposis were frequently encountered risk factors in the studied population with SUA, underlining the need for an interdisciplinary approach in the management of SUA patients.

The ACT questionnaire represented a consistent tool in managing the evolution of SUA treated with monoclonal antibodies. Biologic therapy contributed to quality of life control of SUA, as observed under all three treatment variants. The pulmonary function was sensitively ameliorated (there was an increase in FEV1 after biologic therapy for all biologic treatments used).

Biologic therapy reduced the severity of symptoms and asthma exacerbations; it improved lung function and control over the disease, with a noted increase in daily quality of life. Increasing the education of severe asthma patients about the benefits of following these guidelines indicated that treatment could be carried out by a multidisciplinary team with the help of the family doctor.

However, a number of patients continue to experience inadequate asthma control, highlighting the need for further research and implementation of early therapeutic strategies. Personalized treatment based on individual patient characteristics and regular assessment of biomarkers is crucial for optimizing the management of severe asthma.

## Figures and Tables

**Table 1:** Evolution of patients on Omalizumab therapy.

OMALIZUMAB	T0	T1	T2	*p* *
**FVC (%)**	88.50 (65.00–102.25)	90.30 (78.65–99.65)	95.40 (74.97–104.57)	0.36
**FEV1 (%)**	67.00 (51.35–99.00)	83.10 (68.50–93.55)	82.65 (71.40–95.02)	0.71
**ITIFF (%)**	74.37 (62.36–85.82)	69.23 (66.33–76.56)	74.05 (70.62–81.60)	0.44
**MEF50 (%)**	31.90 (18.20–98.85)	46.50 (37.40–75.25)	53.15 (46.65–78.32)	0.71
**ACT SCORE**	14.0 (12.5–15.75)	19.00 (16.50–20.50)	22.50 (20.50–23.75)	0.04
**WBC COUNT (cells/microliter)**	8565.00 (6025.00–11,600.00)	6560.00 (4620.00–8500.00)	5550.00 (4625.00–7225.00)	0.36
**EOSINOPHILS** **(cells/microliter)**	100.00 (52.50–215.00)	490.00 (80.00–900.00)	350.00 (112.50–625.00)	0.60
**I** **gE (ng/mL)**	603.90 (485.47–784.05)	208.50 (116.97–311.95)	95.50 (45.35–121.35)	0.01

* Friedman’s test. Bold was used to highlight statistical significance.

**Table 2:** Evolution of patients on Benralizumab therapy.

BENRALIZUMAB	T0	T1	T2	*p* *
**FVC (%)**	64.60 (55.90–85.82)	83.15 (73.40–98.00)	85.00 (81.00–107.60)	0.04
**FEV1 (%)**	55.20 (45.32–66.62)	80.00 (67.00–92.00)	77.00 (61.00–102.00)	0.25
**ITIFF (%)**	67.40 (58.34–74.52)	73.00 (68.52–77.00)	65.25 (53.40–78.26)	0.60
**MEF50 (%)**	30.75 (21.50–48.25)	53.00 (35.00–97.00)	50.00 (22.00–78.00)	0.25
**ACT SCORE**	11.50 (8.75–14.25)	19.00 (17.50–20.50)	22.50 (20.25–24.00)	<0.001
**WBC COUNT (cells/microliter)**	7955.00 (6814.75–9695.00)	6410.00 (5763.50–7468.50)	6542.50 (3955.00–8382.50)	0.02
**EOSINOPHILS** **(cells/microliter)**	600.00 (424.00–690.00)	0.00 (0.00–28.50)	0.00 (0.00–14.25)	<0.001

* Friedman’s test. Bold was used to highlight statistical significance.

**Table 3:** Evolution of patients on Dupilumab therapy.

DUPILUMAB	T0	T1	T2	*p* *
**FVC (%)**	55.50 (40.65–68.25)	69.00 (51.95–89.50)	72.50 (52.45–91.50)	0.17
**FEV1 (%)**	54.20 (34.25–59.07)	59.22 (45.20–72.72)	67.70 (38.00–86.50)	0.17
**ITIFF (%)**	64.30 (50.97–80.14)	72.05 (66.35–75.05)	62.80 (51.11–73.34)	0.77
**MEF50 (%)**	27 (15.72–40.47)	40.00 (24.80–47.70)	22.80 (16.50–52.20)	0.47
**ACT SCORE**	10.50 (8.50–13.25)	18.00 (16.00–21.00)	22.00 (19.50–24.00)	<0.01
**WBC COUNT (cells/microliter)**	7570.00 (6450.00–10,817.50)	7720.00 (7400.00–8400.00)	7430.00 (3575.00–9745.00)	0.81
**EOSINOPHILS** **(cells/microliter)**	540.00 (255.00–1030.00)	100.00 (12.00–230.00)	200.00 (90.00–940.00)	0.24

* Friedman’s test. Bold was used to highlight statistical significance.

## Data Availability

Dataset available on request from the authors.
